# Special Announcement

**Published:** 1890-07

**Authors:** 


### Editorial.

### SPECIAL ANNOUNCEMENT.

WITH this number, Dr. W. X. Sudduth will close his connection with this journal as Editor and Business Manager. This change has been rendered necessary by Dr. Sudduth's appointment as Professor of Pathology and Oral Surgery and Secretary of the Dental Faculty in the University of Minnesota. The transfer of the *Independent Practitioner* to the International Dental Publication Company was principally due to the energy of Dr. Sudduth, and his editorial work has been successful in establishing the Journal upon a satisfactory foundation. The success of the *Independent Practitioner* clearly demonstrated that the profession needed an organ free from entangling influences. This the INTERNATIONAL has endeavored to supply. The editor's duties have been of an extremely arduous character, and it is due to him to say that he has proved that a journal, such as this, can be maintained by the dental profession. The support that has been given it in many directions has been very gratifying to the management, and we have no doubt but that this will be generously continued. The past is an earnest of what the future will be under the new management. It is not proposed to make any immediate change in its present satisfactory appearance; but improvements will be introduced as time and necessity seem to suggest and demand.

The Board of Management, in consequence of the unexpected resignation of the Editor, has been obliged to fill the position temporarily, and have selected the chairman of the Advisory Committee, Dr. James Truman, to fill the vacancy. His duties will commence with the August issue of the journal.

ADVISORY COMMITTEE.

